# Interactions Between e-Cigarette Use and Quit Intentions on Cigarette Smoking in Lesbian, Gay, Bisexual, Transgender, Queer, and Other Non-Heterosexual or Cisgender Individuals

**DOI:** 10.1093/ntr/ntaf263

**Published:** 2025-12-24

**Authors:** Lucy A Schuler, Matthew G Kirkpatrick, Shirlene D Wang, Anna Miner, Jimi Huh, Raina D Pang

**Affiliations:** Department of Population and Public Health Sciences, University of Southern California, Los Angeles, CA, USA; Department of Population and Public Health Sciences, University of Southern California, Los Angeles, CA, USA; Department of Psychology, University of Southern California, Los Angeles, CA, USA; Knight Cancer Institute, Oregon Health & Science University, Portland, CA, USA; Department of Population and Public Health Sciences, University of Southern California, Los Angeles, CA, USA; Department of Population and Public Health Sciences, University of Southern California, Los Angeles, CA, USA; Department of Population and Public Health Sciences, University of Southern California, Los Angeles, CA, USA; Department of Psychology, University of Southern California, Los Angeles, CA, USA

## Abstract

**Introduction:**

Anecdotally, e-cigarette use during a cigarette quit attempt is a common quit strategy, but its efficacy is unclear. This study examined e-cigarette use and setting a daily quit intention on cigarette smoking in lesbian, gay, bisexual, transgender, queer, and other non-heterosexual or cisgender individuals, a population with higher rates of both cigarette and e-cigarette use.

**Methods:**

Lesbian, gay, bisexual, transgender, queer, and other non-heterosexual or cisgender individuals in California (*n* = 207, 68% female sex, M[SD] age = 36.3[9.7] years old) completed 35 days of Ecological Momentary Assessments during an unassisted cigarette quit attempt. Each morning, they reported whether they intended to abstain from cigarettes that day (ie, setting a daily quit intention), and each night reported whether they used e-cigarettes and the number of cigarettes smoked that day. Multilevel models tested main effects of current e-cigarette use status and day-level e-cigarette use, as well as interactions with quit intention, on the number of cigarettes smoked.

**Results:**

Fifty-nine percent of participants used e-cigarettes during the study. Those who used e-cigarettes smoked significantly fewer cigarettes compared to those with no e-cigarette use (*p*=.033). Among those who vaped, there was a day-level e-cigarette use $\times$quit intention interaction (*p* < .001). On days with no quit intention, e-cigarette use was associated with more cigarettes smoked. On days with a quit intention, e-cigarette use that day was associated with fewer cigarettes smoked.

**Conclusion:**

While e-cigarette users overall smoked fewer cigarettes compared to those who do not use e-cigarettes, the effects of day-level e-cigarette use on cigarette smoking depend on an individual’s intention to quit that day. e-cigarettes may help smoking cessation, but only if there is an established quit intention.

**Implications:**

Within this sample of LGBTQ+ cigarette smokers engaged in an unassisted quit attempt, the effects of e-cigarette use on cigarette smoking depend on intent to quit that day. These results may help explain previous mixed findings in the literature regarding e-cigarette use on smoking cessation.

## Introduction

Cigarette smoking remains the leading cause of preventable death and disease in the United States.^1^ The majority of adult smokers want to quit;^2^^,^^3^ however, fewer than 10% report successfully quitting or abstaining for at least 6 months.^3^ While the gold standard treatment approach remains a combination of pharmacotherapy and behavioral counseling, it is important to recognize that most quit attempts (approximately 72%) are “unassisted”^2^^,^^3^ (ie, without behavioral or pharmacological treatment) and often spontaneous (ie, started without a previously set quit date).^4^ Given the preponderance of unassisted quit attempts, it is critical to better understand the methods individuals use on their own that may help or hinder their cessation.

One potential method to quit smoking cigarettes could be using e-cigarettes, which have been promoted by the tobacco industry as efficacious and have been reportedly used by many as a smoking cessation aid.^5^^,^^6^ It has been suggested that e-cigarettes could either serve as another form of nicotine replacement therapy or a substitution of sensorimotor stimuli similar to combustible cigarettes.^7^^,^^8^ However, to date, the evidence regarding e-cigarette efficacy for smoking cessation has been mixed. On the one hand, evidence from randomized controlled trials suggests that e-cigarettes may be more effective than traditional nicotine replacement or behavioral interventions,^9^ and recent findings from nationwide longitudinal cohort studies (eg, Population Assessment of Tobacco and Health Study) have shown that daily e-cigarette use (as opposed to non-daily use) is associated with higher rates of cigarette cessation.^10^ However, other studies have been either inconclusive or have shown that e-cigarette use is associated with *reduced* cessation of combustible cigarettes, and even increased smoking and relapses among former smokers.^11–14^ Thus, to date, the efficacy of using e-cigarettes as a cessation tool remains unclear.

There are several methodological factors that could contribute to the above mixed findings. Many of the positive findings come from randomized controlled trials directly examining specific e-cigarette products in the context of formal smoking cessation attempts.^15^^,^^16^ By contrast, many of the negative findings come from observational studies that include participants who may or may not be trying to quit (and/or with unknown quit motivation), and who may use a diverse set of e-cigarette-related products with varying features, including nicotine concentrations, flavors, and device voltage.^12^^,^^13^ Additionally, most previous studies have not taken into account the nuances of daily life during an unassisted quit attempt. For instance, motivation to quit is often operationally defined as a fixed construct (ie, assuming one is always planning to quit), rather than fluctuating over short periods of time. Fluctuations in motivation to quit smoking are common among smokers,^17^ and recent Ecological Momentary Assessment (EMA) studies have found that day-by-day changes in motivation and self-efficacy are associated with smoking outcomes.^18^^,^^19^ It is possible that e-cigarettes may be especially efficacious on days when individuals are motivated to abstain from smoking, but not on days when they do not intend to abstain.

LGBTQ+ (lesbian, gay, bisexual, transgender, queer, and other non-heterosexual or cisgender) individuals have higher rates of both cigarette and e-cigarette use compared to the general population.^20–23^ This may be due to a variety of reasons, including experiencing minority stress,^24–26^ targeted marketing from both cigarette and e-cigarette companies,^27^ and limited cessation resources.^26^ It is also important to note that despite these disparities, sexual and gender minorities do not differ from their non-LGBTQ+ counterparts in desire or motivation to quit smoking cigarettes,^28^^,^^29^ and thus it is important to understand potential methods for smoking cessation among this population. Currently, there is a dearth of information about several cessation-related factors in LGBTQ+ individuals who smoke, including how e-cigarette use affects cigarette smoking and how this association may be impacted by daily intentions to abstain or not.

The current report is a secondary analysis of the day-level data from an EMA study examining cigarette smoking during an unassisted practice quit attempt in same-sex and/or same-gender couples in California. For this analysis, we examined whether: (1) individuals with Current e-Cigarette Use (ie, those who reported use at baseline and/or any use during EMA) smoked fewer cigarettes on average compared to those who did not report e-cigarette use; (2) among those with Current e-Cigarette Use, Day e-Cigarette Use (reported e-cigarette use on that day during EMA) was associated with fewer cigarettes smoked; and (3) reporting a Day Quit Intention in the morning would moderate this association (ie, days with e-cigarette use would be associated with fewer cigarettes smoked especially on days with a quit intention).

## Materials and Methods

### Participants

Participants responded to online advertisements for LGBTQ+ individuals and their partners to participate in a research study (individual *N* = 207, dyad *N* = 103; from March 2021 to January 2023). To be eligible for the parent study, participants had to (1) report smoking at least one cigarette/day for the past year, (2) be in a same-sex or same-gender romantic relationship for at least 6 months with no intentions of separating in the next 35 days with a partner who also meets eligibility criteria and participates in the study, (3) be interested in quitting smoking (ie, respond “yes” to “Do you want to quit or have you been thinking about quitting smoking cigarettes?”), (4) be willing to make a practice quit attempt (ie, respond “yes” to “Would you be willing to make a ‘practice’ quit attempt for the purpose of a study?”), (5) be at least 18 years old, (6) live in California, and (7) have the ability to complete study tasks, including completing videoconference/phone interviews and having a smart device compatible with the LifeData app (eg, iOS or Android), used for EMA data collection (https://www.lifedatacorp.com/). Participants with baseline data and at least 1 day of complete EMA data were included in the current study.

### Procedure

Participants were screened for eligibility based on an online survey filled out by one partner with information about the couple. Both partners then provided informed consent during a phone call with study staff. Once consented, both partners completed an online survey assessing baseline measures before attending the first of two phone/videoconference interviews. During the first interview, study staff assessed smoking history, including any past or current e-cigarette use, prior quit attempts, potential triggers, and motivation to quit cigarette smoking, in preparation for an unaided cigarette quit attempt. These interviews were conducted with each member of the couple separately before training and subsequently starting the 35-day EMA period. During EMA, participants received five surveys per day: one in the morning (WAKE; response window from 5:00 AM to 11:00 AM ), three throughout the day (DAILY; 1-hour response window each, at 11:00 AM, 3:00 PM, and 7:00 PM), and one at night (NIGHT; response window from 8:30 PM to 11:59 PM). The current analysis only includes data from two EMA surveys: the WAKE survey (when that day’s Quit Intention was assessed; response window from 5:00 AM to 11:00 AM) and the NIGHT survey (when that day’s Cigarette Smoking and e-Cigarette Use were assessed; response window from 8:30 PM to 11:59 PM). Participants were paid once a week depending on their survey completion rates, with the opportunity to earn up to $350. All procedures were approved by the University of Southern California Institutional Review Board.

### Measures

#### Baseline Measures

##### Demographics

REDCap electronic data capture tools hosted at the University of Southern California were used to administer baseline measures. An investigator-constructed personal history questionnaire assessed basic demographics including sex (“What sex were you assigned at birth, on your original birth certificate?” with options “Male” and “Female”) and gender identity (“What is your current gender identity? [check all that apply]” with response options “Man”, “Woman”, “Transgender man”, “Transgender woman”, “Genderqueer/gender non-conforming”, and “Different identity [please specify]”).

##### Smoking Characteristics

Cigarette dependence was measured continuously using the Fagerström Test for Cigarette Dependence which scores dependence on a scale from 0 (very low) to 10 (very high).^30^^,^^31^ Number of cigarettes smoked per day at baseline was assessed by asking participants “In the past 30 days, how many cigarettes have you smoked per day?”.

##### Current e-Cigarette Use

To measure baseline e-cigarette use, participants were asked if they had ever used “electronic cigarettes (e-cigarettes)/JUUL or other vaping device”. If they responded yes, they were prompted to answer, “In the last 30 days, how many total days have you used electronic cigarettes/JUUL or other vaping device?” with response options of 0–30. Those who indicated at least 1 day of e-cigarette use in the past 30-days were coded as Current e-Cigarette Use. In addition, participants with at least 1 day of e-cigarette use during the EMA period (see below) were coded as Current e-Cigarette Use even if they reported no e-cigarette use in the baseline questionnaire. In other words, we coded Current e-Cigarette Use (=1) as any participant who indicated past 30-day e-cigarette use at baseline or at least 1 day of e-cigarette use during EMA, otherwise participants were coded as No e-Cigarette Use (=0).

### EMA Measures

#### Quit Intention

Day Quit Intention was assessed every morning at the WAKE survey with the question “Do you plan to NOT smoke today?” with response options of “No, I plan on smoking today” (=0) and “Yes, I plan on NOT smoking today” (=1).

#### Cigarette Smoking

Daily number of cigarettes smoked, the primary outcome, was assessed at the NIGHT survey with the question, “How many cigarettes did you smoke today?” with response options of 0–40.

#### Day-Level e-Cigarette Use

Day e-Cigarette Use was assessed at the end of every day with the NIGHT survey, where participants were asked “Today, have you had any of the following substances? Check all that apply. If you did not use any substances below, select none”. Responses included “caffeine”, “marijuana/cannabis/THC”, “e-cigarettes/JUUL/other vaping device”, “non-cigarette tobacco product”, “alcohol”, or “none”. Those who selected “e-cigarettes/JUUL/other vaping device” were then asked to specify whether their vaping device contained “nicotine”, “cannabis”, “CBD”, or “other”. Responses with “nicotine” selected were coded as 1 for Day e-Cigarette Use.

### Data Analysis

Preliminary analyses included reporting demographic statistics and smoking characteristics by Current e-Cigarette Status. To investigate potential differences in demographics and smoking characteristics between those with Current e-Cigarette Use and No e-Cigarette Use, we compared sample descriptives using independent samples *t* tests for continuous variables and chi-square tests for categorical variables. Preliminary analyses also assessed EMA compliance and correlations of compliance with key variables.

To test the effects of Current e-Cigarette Use and Day Quit Intention on cigarettes smoked, multilevel general linear mixed models tested the effects of Current e-Cigarette Use (between-person: Use vs. No Use) and Day Quit Intention (within-person: No Quit Intention vs. Quit Intention) on the number of cigarettes smoked per day.

To test the effects of Day e-Cigarette Use and Day Quit Intention on cigarette smoking among individuals with Current e-Cigarette Use, multilevel models tested the interaction between Day e-Cigarette Use (within-person: No Use vs. Use) and Day Quit Intention (within-person: No Quit Intention vs. Quit Intention). Significant interactions were probed by pairwise comparisons of estimated marginal means using the SPSS /EMMEANS subcommand.

All models controlled for age, sex, cigarette dependence, alcohol and cannabis use that day, and between-subject variation of Day e-Cigarette Use and Day Quit Intention. Multilevel general linear mixed models used the full-information maximum likelihood estimation with the assumption that the data are missing at random. Analyses were conducted in SPSS (Version 29) with the significance threshold set at *p* < .05.

## Results

### Demographic and Smoking Characteristics

See Supplementary File S1 for participant flow chart. Study descriptives for the full analytic sample and by current e-cigarette use are displayed in Table 1. Participants who completed baseline study measures and at least one complete day of EMA (*N* = 207 individuals, 103 dyads) were included in analyses. One dyad had one member with missing baseline data, explaining the uneven individual *N*. Among the 207 individuals in the sample, 121 (58.5%) reported Current e-Cigarette Use (107 reported at baseline, 14 reported at EMA only). There were no significant group differences in demographic and smoking characteristics. Overall, the sample was predominantly female (68.1%) and woman-identifying (55.6%) and racially diverse (55.9% non-White). Participants reported an average of about 12 cigarettes smoked per day at baseline and low-to-moderate cigarette dependence. There were significant differences between Current e-Cigarette Use groups in EMA compliance, with Current e-Cigarette users having lower response rates to EMA prompts (74.6%) compared to those with No Current e-Cigarette Use (81.9%). While use of non-cigarette tobacco products was overall low throughout EMA (1.12% of prompts), those with Current e-Cigarette Use reported significantly more days of use (2.17%) compared to those with No e-Cigarette Use (0.52%). Of the 885 days with reported e-cigarette use, 524 (59.2%) were exclusive to e-cigarettes and no other substances (eg, other tobacco, cannabis, alcohol).

**Table 1:** Sample Descriptives by the Full Sample and e-Cigarette Use Status

	Total *n* = 207 *N* (%)/*M* (*SD*)	No current e-cigarette use *n* = 86 *N* (%)/*M* (*SD*)	Current e-cigarette use *n* = 121 *N* (%)/*M* (*SD*)	Test of difference *p-*value
Age (years)	36.33 (9.74)	37.58 (8.76)	35.44 (10.32)	.12
Sex (assigned at birth)				.63
Female	141 (68.1%)	57 (66.3%)	84 (69.4%)	
Male	66 (31.9%)	29 (33.7%)	37 (30.6%)	
Gender identity				.65
Woman	115 (55.6%)	46 (53.5%)	69 (57.0%)	
Man	47 (22.7%)	22 (25.6%)	25 (20.7%)	
Transgender woman	5 (2.4%)	3 (3.5%)	2 (1.7%)	
Transgender man	3 (1.4%)	1 (1.2%)	2 (1.7%)	
Genderqueer/gender non-conforming	16 (7.7%)	8 (9.3%)	8 (6.6%)	
Other	21 (10.1%)	6 (7.0%)	15 (12.4%)	
Sexual orientation				.19
Gay or lesbian	137 (66.2%)	60 (69.8%)	77 (63.6%)	
Bisexual	39 (18.8%)	11 (12.8%)	28 (23.1%)	
Heterosexual	2 (1.0%)	0 (0.0%)	2 (1.7%)	
Pansexual	16 (7.7%)	9 (10.5%)	7 (5.8%)	
Other	13 (6.3%)	6 (7.0%)	7 (5.8%)	
Race/ethnicity				.25
American Indian, Alaskan Native	1 (0.5%)	1 (1.2%)	0 (0.0%)	
Asian	4 (1.9%)	1 (1.2%)	3 (2.5%)	
Black, African American	26 (12.6%)	10 (11.6%)	16 (13.2%)	
Middle Eastern	1 (0.5%)	1 (1.2%)	0 (0.0%)	
Pacific Islander	4 (1.9%)	1 (1.2%)	3 (2.5%)	
White	93 (44.9%)	46 (53.5%)	47 (38.8%)	
Hispanic/Latino	52 (25.1%)	19 (22.1%)	33 (27.3%)	
Multiracial	26 (12.6%)	7 (8.1%)	19 (15.7%)	
Baseline smoking characteristics				
Cigarettes smoked/day	12.13 (10.30)	12.31 (9.08)	12.01 (11.12)	.84
Cigarette dependence (FTCD)	3.99 (2.16)	3.98 (2.14)	4.00 (2.19)	.94
EMA characteristics^*^				
Compliance	5539 (78.2%)	2841 (81.9%)	2698 (74.6%)	<.**001**

*p*-values <.05 are bolded.

^*^Total column is out of 7080 possible prompts.

FTCD = Fagerström Test for Cigarette Dependence.

### Effects of Current e-Cigarette Use on Daily Cigarette Smoking

Across the entire sample (*n* = 207), there was a main effect of Current e-Cigarette Use (*F*[1, 183.41] = 4.60, *p*=.033), where current e-cigarette users smoked fewer cigarettes (M[SE] = 4.44[.26]) compared to those without e-cigarette use (M[SE] = 5.24[0.30]). Additionally, on days with a reported quit intention, participants smoked fewer cigarettes (main effect of Day Quit Intention; *F*[1, 4591.47] = 2078.14; *p* < .001, M[SE] = 2.20[0.21]) compared to days without a quit intention (M[SE] = 7.47[0.22]).

### Effects of Day e-Cigarette Use and Quit Intention on Daily Cigarette Smoking

**Figure 1:**
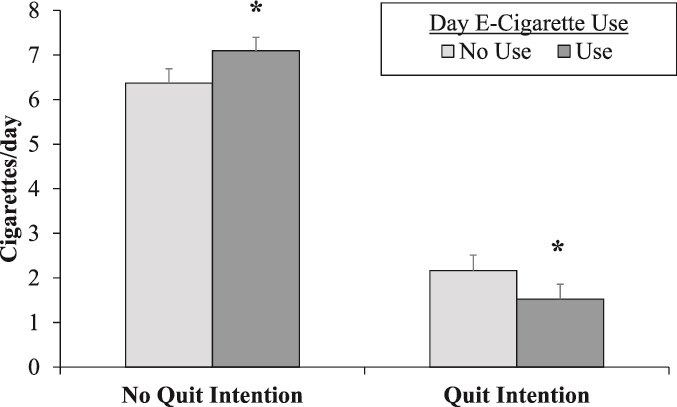
Effects of day e-cigarette use and quit intention on daily cigarette smoking. Note. ^*^ denotes significantly different than no use; *p* < .01.

Among the participants who reported Current e-Cigarette Use (*n* = 121), there was a significant interaction between Day e-Cigarette Use × Day Quit Intention (*F*[1, 2526.19] = 22.46, *p* < .001). Further probing of the significant interaction effects showed that on days with an abstinence intention, e-cigarette use was associated with fewer cigarettes smoked (M[SE] difference = −0.637[.21], *p*=.002). On the other hand, on days without an abstinence intention, e-cigarette use was associated with *more* cigarettes smoked (M[SE] difference = 0.726[.26], *p*=.006). This interaction between Day e-Cigarette Use and Day Quit Intention is shown in Figure 1.

## Discussion

This study examined how e-cigarette use and setting a daily abstinence intention impacted cigarette smoking during a quit attempt in LGBTQ+ individuals. We found that overall, those who reported any current e-cigarette use smoked fewer cigarettes during the study than those without e-cigarette use. Additionally, among e-cigarette users, e-cigarette use that day was only associated with fewer cigarettes smoked on days with a stated quit intention in the morning. Otherwise, e-cigarette use that day was associated with greater smoking. These results suggest that using e-cigarettes may aid smoking cessation in those with a stated intention to abstain that day, but e-cigarette use may be more indicative of dual use if there is no intent to quit that day. These results are consistent with previous research showing the potential efficacy of e-cigarettes as a cessation aid,^9^^,^^10^ while also elucidating potential contexts (eg, daily intentions to abstain or not) that may impact whether e-cigarettes are effective.

Our finding that e-cigarette use is associated with decreased cigarette smoking is consistent with previous research examining e-cigarette use as a cessation tool in randomized controlled trials.^9^ Similar to participants in a randomized controlled trial smoking cessation trial, our participants had at least some motivation to quit (ie, to be included in the study they endorsed willingness to engage in a practice quit attempt). Here, we further extend previous work by demonstrating that motivation to quit (as measured by setting a quit intention) can fluctuate daily, which has implications for daily fluctuations in reduced smoking, whether or not participants were using e-cigarettes. These results also extend previous work by examining naturalistic e-cigarette use with participants using their own devices, which increases external validity. However, a limitation of the current study is that we did not assess which types of devices (or even frequency/intensity of daily use) were used. Additionally, this study did not account for various e-liquids, including whether it was commercially produced or made by the consumer.^32^ Future research should examine the relative impact of different device characteristics on naturalistic smoking behavior during a practice quit attempt. While we only measured 35 days of the practice quit attempt, it is also possible that individuals may use e-cigarettes beyond the attempt, regardless of whether they continue to smoke.^14^ Therefore, the longevity of any positive effects of e-cigarettes on smoking cessation must be considered.

The current results also indicate that among individuals who used e-cigarettes, e-cigarette use on any given day was associated with increased cigarette smoking, but only on days with no stated abstinence intention. This is consistent with previous reports of associations between e-cigarette use and increased cigarette smoking among dual users.^13^ While it makes intuitive sense that e-cigarette use combined with an abstinence intention would decrease smoking, it is not clear from our data why the lack of an abstinence intention and e-cigarette use would increase combustible cigarette smoking. One possibility is that on days with no abstinence intention, participants may have also planned to engage with more opportunities to use nicotine products (eg, when they are not working, or going out for drinks with friends, etc.), and switched between cigarettes and e-cigarettes depending on the appropriateness of the context (eg, some individuals may feel that e-cigarette use is more suitable for indoor contexts).^33^ However, this is speculative as we did not assess the various contexts (or reasons for use) that participants engaged in on a daily basis. While this study focused on the individual level, future research involving this population, especially couples, should also examine how interpersonal factors, like social support, may change and interact with setting a quit intention across the course of a cessation attempt.

These findings must be interpreted in the context of several additional limitations. While we were able to quantify cigarette smoking during EMA, e-cigarette use was only reported as use/no use at the day-level. Therefore, we were unable to ascertain specific e-cigarette use patterns such as flavor, nicotine concentration, device type, and use frequency, which may be predictive of smoking cessation outcomes.^34^ We also controlled for cigarette dependence, rather than more general nicotine dependence which may not account for some patterns of e-cigarette use. While we assessed intentions for cigarette abstinence every day, we did not ask for intentions or motives regarding e-cigarette use. Further research could explicitly investigate motivations for e-cigarette use, especially on a day-to-day level. Additionally, while this study examined intention to abstain in the individual, it is possible that fluctuations in intention are similar in the dyad. Future studies should examine whether these fluctuations are dyadic in nature or whether they are independent of each other.

These results, specifically the interaction between e-cigarette use and setting a quit intention, may provide insight into the mixed findings in the smoking cessation and e-cigarette literature. Prior literature on e-cigarette use has mainly viewed smoking cessation as fixed or static, which may explain the mixed results. Perhaps it’s not necessarily e-cigarette use that promotes smoking cessation, but whether there is intent to quit on a given day. Alternatively, e-cigarette use itself may not be causing increased smoking on days with no abstinence intention, but rather the lack of an abstinence intention produces dual use. Overall, these findings show that when examining the efficacy of e-cigarettes as a cessation aid, day-to-day fluctuations in quit intentions must be considered.

## Supplementary Material

251107_NTR_1_ecig_supp_ntaf263

## Data Availability

The data in this article will be shared upon reasonable request to the corresponding author.
