# Supplementary figures and images for: Interactions Between e-Cigarette Use and Quit Intentions on Cigarette Smoking in Lesbian, Gay, Bisexual, Transgender, Queer, and Other Non-Heterosexual or Cisgender Individuals
Source: Nicotine Tob Res. 2025 Dec 24;28(6):1069–74. doi: 10.1093/ntr/ntaf263 (PMC13196707; doi:10.1093/ntr/ntaf263)

*Supplemental File.* Participant flowchart


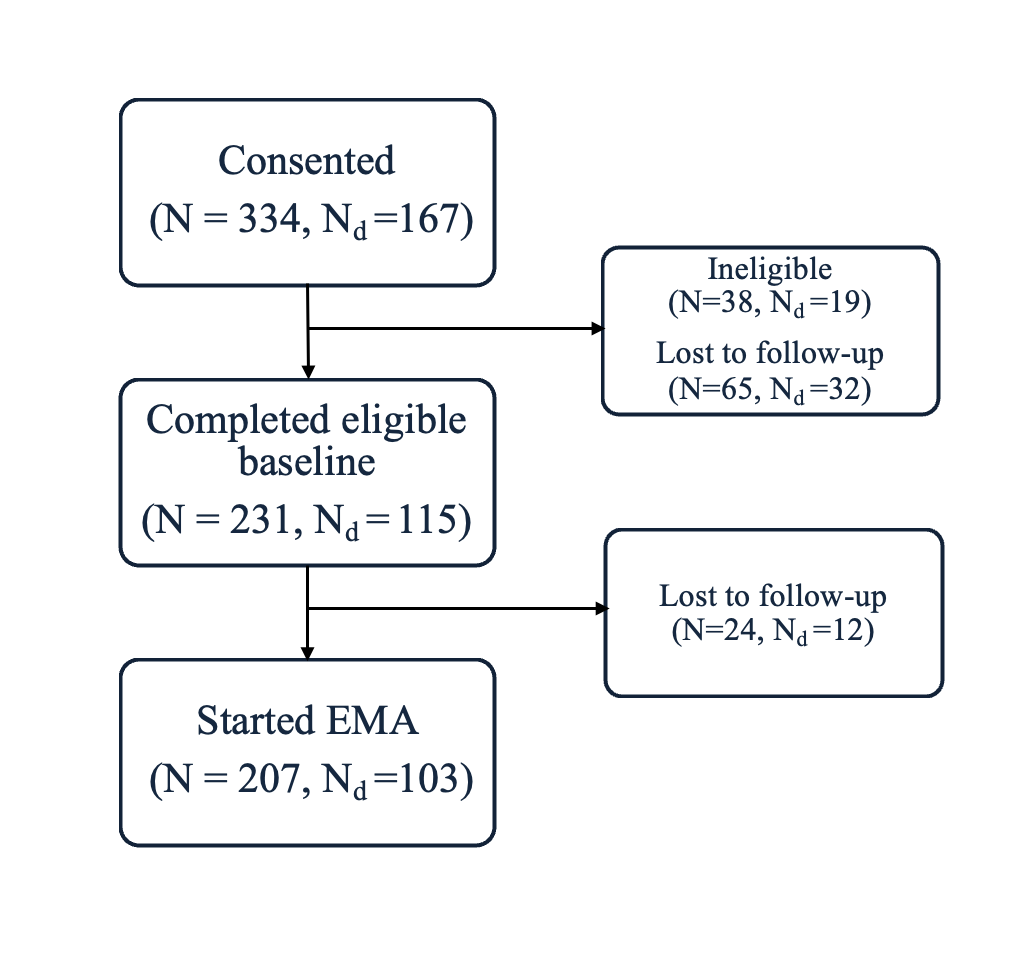

Supplement: 251107_NTR_1_ecig_supp_ntaf263 [file 251107_ntr_1_ecig_supp_ntaf263.docx]
